# Concurrent and Enduring Alignment of Married Partners’ Shared Beliefs and Markers of Aging

**DOI:** 10.1093/geroni/igaa057.2111

**Published:** 2020-12-16

**Authors:** Shannon Mejia, Hannah Giasson, Jacqui Smith, Rich Gonzalez

**Affiliations:** 1 University of Illinois at Urbana-Champaign, Champaign, Illinois, United States; 2 Stanford University, Stanford, California, United States; 3 University of Michigan, Ann Arbor, Michigan, United States

## Abstract

Beliefs about aging are grounded in social experience. This study considers the extent to which married older adults’ shared beliefs about aging and markers of aging maintain a concurrent and enduring association with their partners’ beliefs and markers of aging. Data from the 2010/2012 and 2014/2016 waves of the Health and Retirement Study provided measures of husbands’ and wives’ (3,779 couples) positive and negative beliefs about aging and internal (Cystatin C) and external (grip strength) markers of aging. Latent dyadic models parsed beliefs and markers into partners’ individual and shared variance. Longitudinal analysis showed concurrent associations between shared beliefs and markers of aging to be stable over four years. Meanwhile, the enduring processes that connect beliefs and markers over time were best characterized as bidirectional. The study provides evidence that, within older couples, beliefs about aging are shaped in part through partners’ co-experience of each other’s biological aging.

